# Modeling the volume-effectiveness relationship in the case of hip fracture treatment in Finland

**DOI:** 10.1186/1472-6963-10-238

**Published:** 2010-08-13

**Authors:** Reijo Sund

**Affiliations:** 1 Service Systems Research Unit, National Institute for Health and Welfare, P.O. Box 30, FI-00271 Helsinki, Finland

## Abstract

**Background:**

A common argument in the recent health policy debate is that treatment is more effective among care providers with large volumes. It is challenging, however, to examine the volume-effectiveness relationship empirically. Several suggestions have recently been made for methodological improvements in the examination of the volume-effectiveness relationship. The aim of this study is to develop an extended methodology for examining the volume-effectiveness relationship and demonstrate it for the case of hip fracture treatment.

**Methods:**

Data consisting of 22,857 hip fracture patients from 52 hospitals in Finland in 1998-2001 were extracted from the administrative registers. The relationship between hospital and rehabilitation unit volumes and effectiveness was examined using a statistical model that allowed risk adjustments and hierarchical modeling of volume trends, developed for the purposes of this study. Four-month mortality and the alternative register-based measure of maintainability were used as effectiveness indicators.

**Results:**

No clear relationship was found between hospital volume and the effectiveness of hip fracture treatment, but a novel result showing an association between the rehabilitation unit volume and effectiveness was detected. The face validity of the maintainability indicator seemed to be acceptable.

**Conclusions:**

The methodological ideas presented allow for improved examination of the volume-effectiveness relationship. There are no indications that patients with hip fractures should only be treated in high-volume hospitals, though it may be beneficial to centralize the rehabilitation of hip fracture patients to specialized units.

## Background

A common argument in the recent health policy debate is that treatment is more effective among care providers with large volumes. A wealth of empirical evidence also demonstrates improved effectiveness with selected procedures at high-volume hospitals and by high-volume surgeons [1-4]. It has been suggested that experience or routine (individual and organizational learning), patient selection (better outcomes lead to higher volumes), and the availability of supplementary services (more structure-related resources) may play a part in the relationship between volume and effectiveness, and many of these aspects probably hold true across several health system implementations [5-8]. It has been claimed, however, that the health care provider volume is a nonspecific, indirect, and unreliable measure of provider performance, and a causal relationship between volume and effectiveness has not been proved to exist [9]. In any case, by assuming that the volume-effectiveness relationships are due to human behavior and organizational factors, it is obvious that the interpretations of associations are conditional to the context of observation. In other words, any health policy decision-making related to the volume-effectiveness relationship should be sensitive to potential problems in order to avoid uncritical generalization of international evidence [10].

There have also been methodological drawbacks in the studies that have examined the volume-effectiveness relationship, and several suggestions for methodological improvements have recently been pointed out [11-13]. First, risk adjustment must be considered in the analyses. Second, as the possible volume effect reflects the process of care, some other measure for effectiveness than the most commonly used mortality event - which is a rather crude proxy for effectiveness - should be used [13]. The third issue is to consider the hierarchical nature of the volume effect. While effectiveness should be analyzed at patient level, allowing adequate risk-adjustment, the volume effect must be analyzed at provider level [13]. Moreover, the type of volume relationship (curve-linear, linear, stepwise, cut-off) and the effect of clustering (representing variations in outcomes among providers with similar volumes) should be carefully considered in the model [12,13]. The fourth problem is related to the chance variability of the effectiveness measure. The effectiveness measure (such as mortality) is typically such a rare event that at some providers there may be no or only a few actual events during the observation period. As even one or two events may significantly alter the observed results of low-volume providers, sophisticated hierarchical statistical models should be used that allow conservative shrinkage toward the mean of similar providers [11,13].

### Aim of the study

The aim of this study is to develop an extended methodology for examining the volume-effectiveness relationship. The application of the methodology is demonstrated in the case of hip fracture treatment in the Finnish context by using register-based data.

## Methods

### Setting

In Finland, reliable provider-specific information about the effectiveness of treatments has been considered the only way to monitor the progress of centralization and constitute justified limits for the sizes of practically reasonable units in the Finnish health care system [14].

The organization of social and health care - both of which are incorporated into the same national planning and tax-based financing system - has long been considered a public responsibility in Finland [15]. The country's numerous local authorities - municipalities - are responsible for arranging primary care and other basic services, such as nursing homes and other social services for the elderly [16]. In addition, each municipality is a member of one of the 21 hospital district joint authorities that are responsible for organizing specialized medical services and coordinating hospital treatment in their own districts. Primary health care is mainly provided at health centers that are owned by municipalities or federations of municipalities. The health centers also contain inpatient wards that are mainly used by elderly and chronically ill patients. Secondary and tertiary level medical care is provided by a hierarchy of hospitals, including about forty regional hospitals, sixteen central hospitals, and five university teaching hospitals [17]. Publicly owned hospitals are not run for profit, and there are only a few private hospitals in Finland.

In regard to hip fracture treatment, virtually all hip fracture patients are first referred for examination and surgical treatment to the nearest public hospital with orthopedic services in Finland. After very short postoperative hospital treatment, a hip fracture patient is typically transferred for rehabilitation to the health center [18]. Other services used by hip fracture patients include nursing home care, outpatient health services, and home-help services. Patients have very limited possibilities to choose treatment units, as these are determined based on the patient's municipality of residence.

In the case of hip fracture treatment, there are two volume-related factors that can be regulated fairly easily: the number of orthopedic treatment units and the number of rehabilitation units. The main policy-relevant question can be stated as: Is it possible to improve the effectiveness of hip fracture treatment by regulating the minimum volume for the treatment units?

### Data

In order to examine the volume-effectiveness relationship, data on comparatively risk-adjusted effectiveness indicators are needed for all care providers. The amount of data required is so massive that administrative registers are the only realistic source of such data, in spite of their known shortcomings, such as their secondary nature and the lack of clinical data for risk-adjustment purposes [19,20]. In Finland, very good administrative registers are available, and the personal identification number allows deterministic record-linkage within and between registers. In general, the complete registration, combined with easily linkable registers, makes large, longitudinal population-based studies feasible in Finland [21].

For the purposes of this study, the total population of hip fracture patients in 1998-2001 was identified in the Finnish Health Care Register. The medical histories (1987-2002) and deaths (1998-2002) of the hip fracture population were extracted from the Finnish Hospital Discharge Register, the Finnish Health and Social Welfare Care Register, and the National Causes of Death statistics using the unique personal identification codes of the patient population. Each record in these registers includes data such as patient and provider ID numbers, age, sex, area codes, and diagnosis and operation codes, as well as dates of admission, operation, and discharge (or death). The validity of Finnish register-data for studying the effectiveness of hip fracture treatment is known to be good [22].

Data were pre-processed so that the information concerning hip fracture patients with their first hip fracture could be accurately identified. The details of the process are reported elsewhere [23]. The existence of possible comorbidities was extracted for each patient from his or her medical history using the diagnosis codes recorded in the data. The extraction method was adapted from the Charlson comorbidity categories, and the application to the current data set was done in a similar fashion to that of previous hip fracture studies [24-26]. Other relevant variables available in register-based data, such as age, sex, source of admission, and prior use of care, were also extracted from the data for risk adjustment purposes.

The data set used in this study included data for 22,857 hip fracture patients from 52 hospitals. The volume-effectiveness relationship for rehabilitation units was investigated using a subset of data including hip fracture patients aged 65 years and older who lived at home before the fracture. This subset included 10,384 patients who were transferred to a rehabilitation unit (n = 272) after an operation.

### Effectiveness indicators

While using data from administrative registers, only a limited number of validated effectiveness indicators are available. The most common one is mortality. The use of short-term mortality as an effectiveness measure in volume-effectiveness studies has been criticized, however, because it is a rather crude proxy for effectiveness and also a rather uncommon event that may cause problems in statistical modeling [13]. Moreover, short-term mortality is a weak effectiveness indicator in the sense that many of the perioperative deaths of hip fracture patients may be unavoidable [27]. Four-month mortality was therefore selected as a primary effectiveness indicator in this study. The limit of four months corresponds to the population level maximum for the length of the acute hip fracture treatment episode [28].

There are other possible effectiveness indicators, such as re-hospitalizations or the occurrence of complications. Unfortunately, the indicators that require complex data abstraction using diagnosis codes, such as in the identification of complications, are prone to severe bias caused by existing differences in the registration practices of (secondary) diagnoses. It has been shown, however, that the Finnish register data allow a complete reconstruction of hip fracture treatment episodes in terms of daily levels of care, for which the directly observable levels of care are: 1) home (including home care, ordinary service houses, and outpatient care), 2) nursing home (service houses with 24-hour assistance and residential homes), 3) health center (inpatient ward of local primary care unit), 4) hospital, and 5) death [23]. It is also known that each level of care reflects a certain intensity and need for care [29]. In this sense, it can be interpreted that the directly observable backward steps in the levels of (inpatient) care in the treatment episode following the hip fracture reflect an increased need for care, i.e., obvious drops in the health status of the patient. For the purposes of this study, a new effectiveness measure of maintainability was defined: maintainability can be considered satisfactory if no backward steps are observed in the levels of care. In practice, this measure describes whether there have been some unexpected steps during the treatment (by capturing deaths, readmissions, and referrals to higher-level hospitals). Here, maintainability was operationalized as a dummy variable that indicates unsuccessful maintainability if an event that breaks maintainability was observed during the first four months after the hip fracture.

### Basic model for the volume-effectiveness relationship

The basic idea in volume-effectiveness analyses is to compare the effectiveness of treatment between providers (such as hospitals). This kind of activity is commonly referred to as profiling of providers. Profiling can be quite complicated, as there is variation between providers for at least three reasons: 1) differences may be attributable to random variation due to the size of the provider, 2) the patient case-mix varies from provider to provider, and 3) providers may differ in the effectiveness of their care [30]. For these reasons, a statistical model for provider profiling, in which provider differences are modeled explicitly, must be considered for justified conclusions.

Traditionally, the ratio of observed to expected outcomes multiplied by the mean rate is used as the risk-adjusted rate for providers [31]. In the case of a binary response variable, a logistic regression is a suitable tool for the calculation of expected outcomes. The idea is to construct and estimate a model in which the observed outcome (Y) is a dependent variable and patient characteristics (x) are independent variables. With this kind of model, it is possible to calculate predicted values for all individuals, using patient characteristics and estimated values of parameters with the inverse logit transformation. As the focus of profiling is on providers and not on individuals, the observed and expected outcomes must be aggregated to the provider level as follows:

$${\text{O}}_{\text{i}}=\Sigma \;{\text{Y}}_{\text{j}}$$

and

$${\text{E}}_{\text{i}}=\Sigma {\text{ logit}}^{-\text{1}}({\text{x}}_{\text{j}}\beta )$$

where the sums are over j patients treated by provider i, while β is an estimated parameter vector [32].

As the observed outcomes O_i _are non-negative integers describing frequencies of events, they can be assumed to have a Poisson distribution with an unknown mean μ_i_:

$${\text{O}}_{\text{i}}\sim \text{Poisson}(\mu_{\text{i}})$$

where

$$\text{log }\mu_{\text{i}}={\text{log E}}_{\text{i}}+\theta_{\text{i}}$$

and i is the provider index [33]. In other words, it is assumed that the expected outcomes E_i _adjust the patient characteristics, and θ_i _describes the variation caused by the provider. The use of logarithms guarantees that θ_i _remains positive in this kind of random effects model.

In data sets with a hierarchical structure, there often exist correlations between observations that may result in overestimated differences in profiling analyses. Small sizes of providers may also cause some estimation problems. Assuming exchangeability of providers (i.e., that the results for all providers are equal if there is an infinite number of [similar] patients), a two-level hierarchical model can be used to deal with such problems. A simple solution is to assume that variation caused by providers is normally distributed:

$$\theta_{\text{i}}\sim \text{N}(\alpha ,\sigma^{\text{2}})$$

where exp(α) is the "general" risk-adjusted ratio and σ^2 ^describes the variance between providers [33]. In order to define a full probability model, prior distributions for the parameters α and precision τ = 1/σ^2 ^must also be defined. Suitable non-informative priors are

$$\alpha_{\text{prior}}\sim \text{N}\left(0,\text{1}0^{\text{6}}\right)$$

and

$$\tau_{\text{prior}}\sim \Gamma \left(\text{1}0^{-\text{6}},\text{ 1}0^{-\text{6}}\right).$$

### Extended model for the volume-effectiveness relationship

Hierarchical models, similar to the one presented above, are widely applied in provider profiling and are known to be superior to non-hierarchical models [34]. Unfortunately, the presented model is not optimal for the investigation of a possible relationship between effectiveness and volume because the observations are shrunk towards the global mean, even though it can be hypothesized that there will be some kind of trend between the volume and provider-specific effectiveness measures. In fact, it has been hypothesized that the relationship between volume and effectiveness may be non-linear, linear, stepwise, or may have a single cut-off [13].

In the model presented above, the logarithm of the ratio between observed and expected outcomes was used as a convenient starting point for the model. This means that technically, it would be convenient to incorporate also the possibility of a trend on the logarithmic scale. In fact, the ratio between observed and expected outcomes is a measure of relative difference, and the log difference is the preferred scale for such measures [35]. It is also known that the relative difference approximates to the more adequate log-difference measure in the proximity of ratio one, which means that the interpretations are approximately equal, if the differences are quite small.

The basic model is actually a special case of a linear trend model in which the slope parameter is fixed at zero. The model can be modified in a straightforward way to include the possibility of a volume-related linear trend. More specifically, let z_i _be the provider-specific volume and

$$\theta_{\text{i}}\;\sim \;\text{N}(\alpha_{\text{i}},\sigma^{\text{2}}),$$

where

$$\alpha_{\text{i}}=\alpha +\gamma {\text{z}}_{\text{i}},$$

and priors for α and τ = 1/σ^2 ^are as above, and, correspondingly, a non-informative prior for the slope parameter is

$$\gamma_{\text{prior}}\sim \text{ N}\left(0,\text{ 1}0^{\text{6}}\right).$$

In principle, the same model works in the single cut-off case: if z_i _is changed to a dummy-variable indicating the "high-volume" provider. Similarly, a stepwise model could be implemented by adding regression parameters and dummy variables to the model. The practical problem for the non-continuous models is the determination of appropriate cut points. It is possible to use predetermined limits or try to estimate optimal cut points with the data [36]. With the hierarchical full probability models, it would be possible to build a model for the single cut-off case where the cut-off point is treated as a parameter that is estimated simultaneously with the other parameters. Such a model, however, is not considered here because the estimation easily results in multimodal posterior distributions.

The extension of the model to incorporate a non-linear trend is a little more challenging. The simple parametric approach of using low-order polynomials in the regression model offers only a limited family of shapes and, with more complex forms, it is typically very difficult to choose between well-fitting models. In principle, regression using the fractional polynomial approach could be a satisfactory compromise but would require the fitting of numerous regression models [37]. With the hierarchical modeling approach, it is actually more tempting to use the recently invented connection between penalized splines and linear mixed models to extend the standard regression model to a semi-parametric form in which the non-linear relationship is not restricted by the parametric forms [38]. The aim of such models is to describe the local structure of the relationship between outcome and covariate, resulting in a good fit across the range of the covariate.

The linear model presented above can also be extended to the semi-parametric form. In fact, with a thin-plate spline regression modification, the model remains similar in regard to θ_i_, but α_i _is extended to the form

$$\alpha_{\text{i}}=\alpha +\gamma {\text{z}}_{\text{i}}+\Sigma_{\text{j}=\text{1},...,\text{k}}{\text{b}}_{\text{j}}{\text{w}}_{\text{ij}},$$

where the random coefficients are normally distributed with zero mean and variance σ_b_^2^, i.e.,

$${\text{b}}_{\text{j}}\sim \text{N}(0,\;\sigma_{\text{b}}{}^{\text{2}}),$$

k is the number of so-called knots, and w_ij _are special design variables calculated using k sample quantiles of the covariate [39]. The priors for α, γ, and σ^2 ^are as above, and an adequate non-informative prior for τ_b _= 1/σ_b_^2 ^is

$$\tau_{\text{b}\_\text{prior}}\sim \Gamma \left(\text{1}0^{-\text{6}},\text{1}0^{-\text{6}}\right).$$

### Application of the models

In this study, the three different volume models described above - a mean model, a linear trend model, and a spline model - were applied to the examination of the volume-effectiveness relationship between the four-year (1998-2001) pooled hospital or rehabilitation unit volume and two effectiveness measures, four-month mortality, and maintainability. The predicted probabilities of mortality and maintainability required for risk-adjustment purposes were estimated using the logistic regression model, and the predictive power of the model was measured using the c-statistics. The hierarchical models were estimated using MCMC simulation. Five knots were used in the specification of the spline model. The mixing of the estimation procedure was examined using two chains in the estimation, and the convergence was evaluated on the basis of Gelman-Rubin convergence plots [40]. A hundred thousand iterations following ten thousand burn-in iterations were used in the actual estimation of the parameters for each model. The complexity and relative fit of the hierarchical models were assessed with the deviance information criterion (DIC) [41].

## Results

The basic characteristics for all hip fracture patients in Finland in 1998-2001 and for patients aged 65 years and older who lived at home before the fracture and who were treated in a rehabilitation unit after surgical admission are presented in Table 1. They appear to be very similar regardless of the obvious differences in age, proportion of men, and care history.

**Table 1:** Basic characteristics and factors predicting four-month mortality and maintainability among hip fracture patients in Finland, years 1998-2001

	All hip fracture patients %	Four-month mortality OR (95% CI)	Four-month unsuccessful maintainability OR (95% CI)	Patients in rehabilitation units* %	Four-month mortality OR (95% CI)	Four-month unsuccessful maintainability OR (95% CI)
Number of observations	22,857	22,857	22,857	10,384	10,384	10,384

Number of events		4,299	9,991		1,714	4,833

Demographic variables						
Age** (mean)	77.4	1.91 (1.83 to 2.00)	1.21 (1.19 to 1.24)	81.1	2.04 (1.87 to 2.22)	1.27 (1.20 to 1.35)
Men	29.7	2.11 (1.95 to 2.28)	1.50 (1.41 to 1.60)	24.6	1.97 (1.74 to 2.22)	1.52 (1.39 to 1.67)
Type of fracture						
Neck of femur	63.2	1	1	62.1	1	1
Trochanteric	30.0	1.01 (0.94 to 1.09)	0.93 (0.87 to 0.98)	31.3	1.00 (0.89 to 1.13)	0.89 (0.82 to 0.97)
Subtrochanteric	6.8	1.12 (0.97 to 1.30)	1.03 (0.92 to 1.15)	6.8	1.07 (0.86 to 1.33)	0.90 (0.76 to 1.05)
Care history						
Long-term care patient	17.8	1.02 (0.93 to 1.12)	0.60 (0.55 to 0.65)	-	-	-
Recent short-term care	23.6	1	1	13.3	1	1
No recent care	58.6	0.46 (0.43 to 0.50)	0.54 (0.51 to 0.58)	86.7	0.40 (0.35 to 0.46)	0.58 (0.51 to 0.65)
Comorbid conditions						
Cancer	11.3	1.68 (1.53 to 1.86)	1.46 (1.34 to 1.59)	12.2	1.77 (1.53 to 2.05)	1.47 (1.30 to 1.66)
Diabetes	10.1	1.33 (1.19 to 1.48)	1.40 (1.28 to 1.53)	10.6	1.25 (1.06 to 1.48)	1.40 (1.23 to 1.60)
Parkinson's disease	2.9	1.14 (0.95 to 1.38)	1.30 (1.11 to 1.53)	3.0	1.22 (0.90 to 1.65)	1.55 (1.23 to 1.97)
Cardiovascular disease	34.2	1.50 (1.39 to 1.61)	1.38 (1.30 to 1.47)	36.9	1.32 (1.18 to 1.48)	1.28 (1.17 to 1.39)
Cerebrovascular disease	18.9	1.14 (1.05 to 1.24)	1.15 (1.07 to 1.23)	18.2	1.24 (1.08 to 1.41)	1.22 (1.10 to 1.36)
Peripheral vascular disease	4.5	1.20 (1.04 to 1.40)	1.28 (1.12 to 1.46)	4.9	1.56 (1.25 to 1.95)	1.55 (1.28 to 1.87)
Chronic pulmonary disease	8.5	1.24 (1.11 to 1.39)	1.30 (1.18 to 1.44)	9.3	1.27 (1.06 to 1.51)	1.32 (1.15 to 1.52)
Peptic ulcer disease	3.7	1.08 (0.91 to 1.28)	1.25 (1.08 to 1.44)	4.0	1.17 (0.90 to 1.52)	1.28 (1.04 to 1.56)
Renal disease	0.9	2.05 (1.52 to 2.76)	1.98 (1.47 to 2.67)	0.9	1.83 (1.15 to 2.91)	1.84 (1.18 to 2.86)
Rheumatologic disease	5.3	0.96 (0.81 to 1.13)	1.21 (1.07 to 1.36)	6.1	0.99 (0.78 to 1.25)	1.34 (1.14 to 1.59)

C-statistics		0.74	0.65		0.71	0.63

OR = Odds Ratio, CI = Confidence Interval

* Among hip fracture patients aged 65 years and older who lived at home at the time of fracture

** Odds ratio per ten years increase in age

The average four-month mortality among all hip fracture patients was 18.8% and the average unsuccessful maintainability was 43.7%. Of the 9,991 first events of unsuccessful maintainability, 3,275 (32.8%) were deaths, 3,522 (35.3%) readmissions, and 3,194 (32.0%) referrals to higher-level providers. The corresponding figures for the subset of patients treated in rehabilitation units were 16.5% for mortality and 46.5% for unsuccessful maintainability, and of the 4,833 first events of unsuccessful maintainability, 1,153 (23.9%) were deaths, 1,754 (36.3%) were readmissions, and 1,926 (39.9%) were referrals to higher-level providers.

The odds ratios from the logistic regression models used in risk adjustment are also reported in Table 1. The effects of age and sex were stronger in the mortality models than in the maintainability models. Comorbid conditions had a tendency to slightly stronger effects in the maintainability models than in the mortality models except for renal and vascular diseases, and cancer. Somewhat surprisingly, variables indicating trochanteric fracture and the status of long-term care patient had a protective effect in the maintainability models.

**Figure 1:**
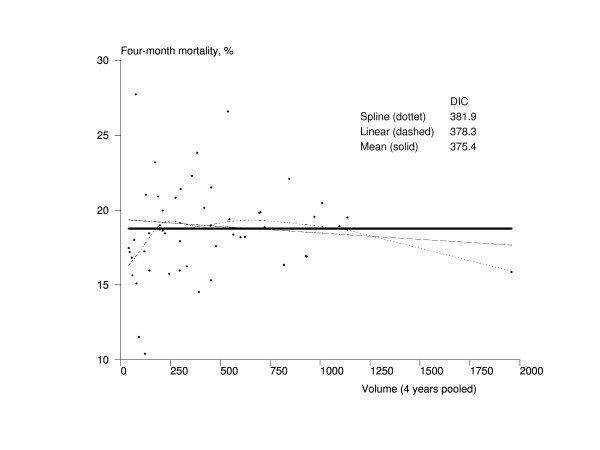
**Association between the volume of the hospital and mortality among Finnish hip fracture patients in 1998-2001**. The x-axis represents the volume of the pooled number of treated hip fracture patients in hospital during 1998-2001 in Finland, and the y-axis contains the four-month risk-adjusted mortality. The dots represent hospitals (n = 52). The solid line is the trend from the mean model, the dashed line is the trend from the linear model, and the dotted line is the trend from the spline model. DIC = Deviance Information Criterion

**Figure 2:**
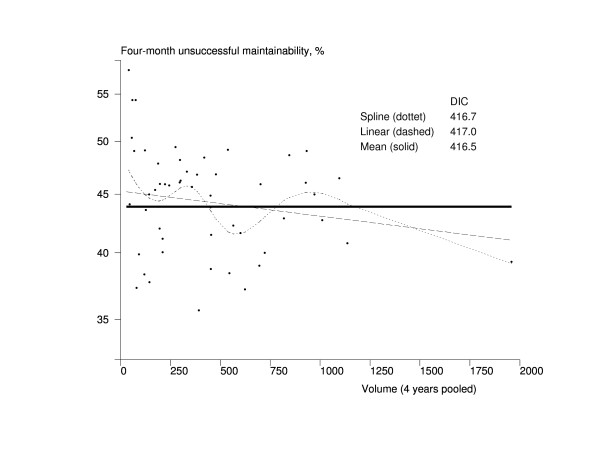
**Association between the volume of the hospital and maintainability among Finnish hip fracture patients in 1998-2001**. The x-axis represents the volume of the pooled number of treated hip fracture patients in hospital during 1998-2001 in Finland, and the y-axis contains the four-month risk-adjusted unsuccessful maintainability (death, readmission, or referral to a higher-level hospital). The dots represent hospitals (n = 52). The solid line is the trend from the mean model, the dashed line is the trend from the linear model, and the dotted line is the trend from the spline model. DIC = Deviance Information Criterion

**Figure 3:**
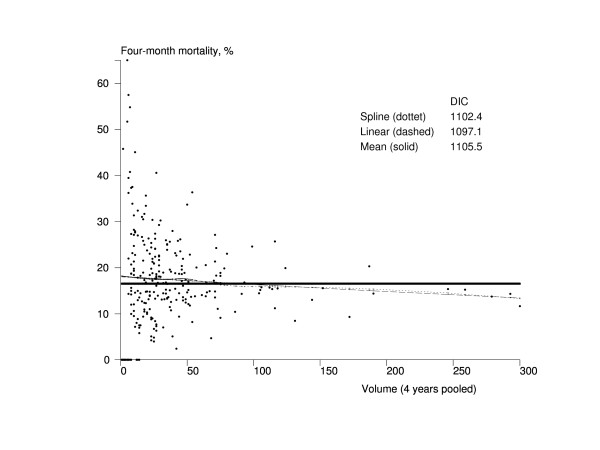
**Association between the volume of the rehabilitation unit and mortality among Finnish hip fracture patients aged 65 years and older who lived at home before the fracture in 1998-2001**. The x-axis represents the volume of the pooled number of treated hip fracture patients in a rehabilitation unit during 1998-2001 in Finland, and the y-axis contains the four-month risk-adjusted mortality. The dots represent rehabilitation units (n = 272). The solid line is the trend from the mean model, the dashed line is the trend from the linear model, and the dotted line is the trend from the spline model. DIC = Deviance Information Criterion

**Figure 4:**
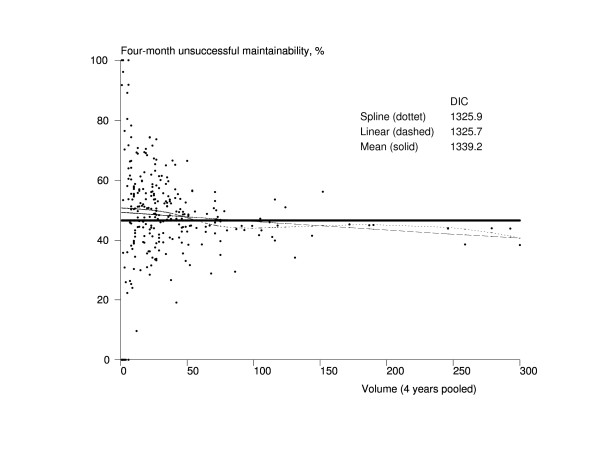
**Association between the volume of the rehabilitation unit and maintainability among Finnish hip fracture patients aged 65 years and older who lived at home before the fracture in 1998-2001**. The x-axis represents the volume of the pooled number of treated hip fracture patients in a rehabilitation unit during 1998-2001 in Finland, and the y-axis contains the four-month risk-adjusted unsuccessful maintainability (death, readmission, or referral to a higher-level hospital). The dots represent rehabilitation units (n = 272). The solid line is the trend from the mean model, the dashed line is the trend from the linear model, and the dotted line is the trend from the spline model. DIC = Deviance Information Criterion

The results of the volume-effectiveness association models are presented in Figures 1, 2, 3 and 4. The hospital volume had no association whatsoever with four-month mortality, and the mean model was obviously the best fitting one according to DIC (Figure 1). Based on Figure 2, there seemed to be a trend towards better maintainability in high-volume hospitals. The mean model had a better fit, however, according to the DIC (416.5) compared with the DIC of the linear trend model (417.0). The spline model also had a smaller DIC value (416.7) than the linear model, but the shape of the trend was very complex, indicating that the mean model was also the most appropriate one in this case.

The volume of the rehabilitation unit was linearly associated with four-month mortality, and larger units were more effective (Figure 3). The trend of the spline model had a similar shape to that of the linear model but, being more complex model, its DIC (1102.4) was bigger than the one from the linear model (1097.1). A clear association was also found between the volume of the rehabilitation unit and four-month maintainability (Figure 4). The linear model and the spine model had almost the same DIC (1325.7 vs. 1325.9), but the spline model indicated that the association could be a cut-off type rather than linear so that the units treating about 25 or more hip fracture patients per year would have better results.

## Discussion

In this study, the volume-effectiveness relationship was examined from the methodological point of view. Recent suggestions for methodological improvements in volume-effectiveness studies could be summarized as a need for: 1) hierarchical modeling that allows risk adjustment at patient level and examination of volume effect at provider level, so that clustering and different types of volume relationships (curvelinear, linear, stepwise, cut-off) can be taken into account; and 2) an effectiveness measure that is not as rare an event as short-term mortality and that also reflects the process of care [11-13]. In this study, a methodological approach that aimed to fulfill both of these needs was developed in tandem with examining the volume-effectiveness relationship in the case of hip fracture treatment using Finnish register data.

Several studies have previously examined the volume-effectiveness relationship in the case of hip fracture treatment, but the results have been mixed [42-60]. In the current study, no volume effect was found between the hospital volume and effectiveness in terms of mortality, and there was only a weak tendency for positive association in terms of maintainability. These results are in line with the previous Finnish hip fracture study, which did not find any volume effect on mortality or acute complications [42]. As a conclusion of the international studies, in most cases there has only been a weak trend toward greater effectiveness with higher volumes of treated hip fracture patients, and it is likely that the feasible improvements in effectiveness related to the surgeon or hospital volume are negligible compared with the unavoidable major adverse outcomes related to the hip fracture condition itself [43-60].

More interestingly, by focusing on the volume of the rehabilitation unit, there was a clear positive volume effect with both effectiveness indicators used in this study. This was a novel finding, but not a surprising one, as volume-effectiveness associations have been found in nursing home care [61], and it is well known that adequate rehabilitation of hip fracture patients improves effectiveness significantly [62,63]. The exact mechanisms behind the detected relationship cannot be explained in this study, but it is likely that effectiveness is simply worse if there is no routine for hip fracture treatment. The structure-related resources and organizational learning probably also have a major, but indirect, role in the sense that the whole process of care tends to be better for providers that have greater availability of support services, possibilities for specialization, and enough resources for continuous improvements in care practices.

In regard to data sources, there are not many options for administrative registers while studying the volume-effectiveness relationship. The selection of hip fractures as the health problem of interest had certain advantages in a study using administrative data: it is a relatively common disease (enough data and relevant from the policy point of view); it is quite easy to diagnose (can be accurately identified from the registers); virtually all hip fracture patients are treated in hospital (all patients can be found from the registers); and it was possible to observe detailed treatment pathways of these, typically, elderly patients using the Finnish register data [23]. The validity of the data was also known to be very good [22].

Two effectiveness indicators were used in this study: mortality and maintainability. Mortality is a well-established and commonly used effectiveness indicator that objectively captures the most serious adverse outcome. In this study, four-month mortality was used as a yardstick for the alternative maintainability indicator.

Maintainability was defined as a backward step in the levels of care, i.e., in terms of events that were robustly and completely identifiable from the register data. By capturing deaths, readmissions, and referrals to higher-level providers, the event is far more frequent than short-term mortality. It also captures more from the care process than only the death events. More complex events are likely to be harder to predict using the available background factors in the adjustment, so it was expected that the predictive power of the maintainability models was lower than of the mortality models measured using the c-statistics (Table 1). The c-statistics of the maintainability models remained at the level that is known to be rather typical for hospitalization responses with corresponding background factors [64].

Maintainability seems to reflect the need for care slightly better than mortality in the sense that the effects of age and sex were weaker, and many non-fatal diseases had at least a tendency to a stronger effect than in the mortality models. The protective effect of the variable indicating preceding long-term care in relation to short-term care is probably due to two overlapping reasons: many long-term care patients live in a nursing home and all their problems do not necessarily result in the need for upper-level care, and there are simply fewer upper levels of care for long-term care patients than for the patients coping at home. The protective effect of trochanteric fractures in relation to fractures of the neck of the femur may be related to differences in treatment practices of intra- and extracapsular hip fractures [65].

The face validity of the maintainability indicator seemed to be acceptable in this study. The interpretations of volume-effectiveness associations turned out to be quite similar to mortality, although maintainability added more details to the associations. The main drawback of the maintainability measure was that it was not specific to the health problem of interest: All backward steps in the levels of care were considered adverse outcomes regardless of the actual reasons. It also seems that the interpretations can be strengthened by restricting the analyses to subpopulations that are homogeneous in terms of possible transitions between levels of care, such as elderly hip fracture patients living at home at the time of fracture. In any case, the maintainability measure seems to reflect quite adequately whether everything has gone smoothly during the treatment process of elderly patients at the population level, as long as the potential restrictions are kept in mind.

In this study, an extended methodological approach that allows risk adjustment and hierarchical modeling of volume trends was developed. The aim was to diminish the recognized biases attributable to the use of more traditional methods. As such, the improved methodology presented in this study should be useful for further examinations of the volume-effectiveness relationship.

It must be noted, however, that the presented models were not perfect, and the approach was intended to study associations, not causality. Due to the limitations of the data, an additional level for surgeons was omitted from the models. The surgeon level would have been particularly interesting when studying hospital volumes, as patients are obviously clustered within surgeons and surgeons within hospitals (although surgeons may operate in a number of different institutions depending on the local health care configuration). In addition, another level could be incorporated to capture the variation attributable to the operative team rather than just the surgeon. On the other hand, the utility of additional levels for studying the volumes of rehabilitation units seems not to be as obvious. It is also likely that strong volume associations can be detected with simpler models, and the more complex ones may then be used to confirm and possibly explain the existing relationships. Other possible methodological development lines for further studies include the implementation of risk adjustment and volume association models as one model, relaxation of the Poisson assumption, incorporation of a more detailed variance structure, and models for responses other than binary ones.

## Conclusions

The improved methodology presented in this study should be useful for examinations of the volume-effectiveness relationship in fairly general cases. In the current hip fracture case study, no clear relationship was found between hospital volume and effectiveness. However, for the first time ever, an association was detected between the volume of the rehabilitation unit and effectiveness. There are no indications that patients with hip fractures should only be treated in high-volume hospitals, but it may be beneficial to centralize the rehabilitation of hip fracture patients to specialized units.

## Competing interests

The author declares that he has no competing interests

## Authors' contributions

RS carried out the whole research process.

## Pre-publication history

The pre-publication history for this paper can be accessed here:

http://www.biomedcentral.com/1472-6963/10/238/prepub

## References

[B1] Dudley RA Johansen KL Brand R Rennie DJ Milstein A Selective referral to high-volume hospitals: estimating potentially avoidable deaths Jama 2000 283 9 1159 1166 10.1001/jama.283.9.1159 10703778

[B2] Halm EA Lee C Chassin MR Is volume related to outcome in health care? A systematic review and methodologic critique of the literature Ann Intern Med 2002 137 6 511 520 12230353 10.7326/0003-4819-137-6-200209170-00012

[B3] Sowden A Aletras V Place M Rice N Eastwood A Grilli R Ferguson B Posnett J Sheldon T Volume of clinical activity in hospitals and healthcare outcomes, costs, and patient access Qual Health Care 1997 6 2 109 114 10.1136/qshc.6.2.109 10173253 PMC1055462

[B4] Gandjour A Bannenberg A Lauterbach KW Threshold volumes associated with higher survival in health care: a systematic review Med Care 2003 41 10 1129 1141 10.1097/01.MLR.0000088301.06323.CA 14515109

[B5] Posnett J McKee M, Healy J Are bigger hospitals better? Hospitals in a changing Europe 2002 Buckingham: Open University Press 100 118

[B6] Hewitt M Interpreting the volume-outcome relationship in the context of health care quality: Workshop summary 2000 Washington, DC: National Academy of Sciences

[B7] Kraus TW Buchler MW Herfarth C Relationships between volume, efficiency, and quality in surgery - a delicate balance from managerial perspectives World J Surg 2005 29 10 1234 1240 10.1007/s00268-005-7988-5 16136283

[B8] Gandjour A Lauterbach KW The practice-makes-perfect hypothesis in the context of other production concepts in health care Am J Med Qual 2003 18 4 171 175 10.1177/106286060301800407 12934954

[B9] Sheikh K Reliability of provider volume and outcome associations for healthcare policy (with discussion) Med Care 2003 41 10 1111 1128 10.1097/01.MLR.0000088085.61714.AE 14515105

[B10] Phillips KA Luft HS The policy implications of using hospital and physician volumes as "indicators" of quality of care in a changing health care environment Int J Qual Health Care 1997 9 5 341 348 10.1093/intqhc/9.5.341 9394202

[B11] Shahian DM Normand SL The volume-outcome relationship: from Luft to Leapfrog Ann Thorac Surg 2003 75 3 1048 1058 10.1016/S0003-4975(02)04308-4 12645752

[B12] Panageas KS Schrag D Riedel E Bach PB Begg CB The effect of clustering of outcomes on the association of procedure volume and surgical outcomes Ann Intern Med 2003 139 8 658 665 14568854 10.7326/0003-4819-139-8-200310210-00009

[B13] Christian CK Gustafson ML Betensky RA Daley J Zinner MJ The volume-outcome relationship: don't believe everything you see World J Surg 2005 29 10 1241 1244 10.1007/s00268-005-7993-8 16136280

[B14] Duodecim, Finnish Academy Does centralization bring quality to specialized health care? Concensus statement given by the Finnish Medical Society Duodecim and the Finnish Academy [in Finnish] Duodecim 2003 119 4 347 357 12708205

[B15] Häkkinen U Lehto J Reform, change, and continuity in Finnish health care J Health Polit Policy Law 2005 30 1-2 79 96 10.1215/03616878-30-1-2-79 15943388

[B16] Häkkinen U The impact of changes in Finland's health care system Health Econ 2005 14 Suppl 1 S101 118 10.1002/hec.1030 16161191

[B17] Järvelin J Health care systems in transition: Finland 2002 Copenhagen: European observatory on health care systems

[B18] Huusko T Karppi P Avikainen V Kautiainen H Sulkava R Significant changes in the surgical methods and length of hospital stay of hip fracture patients occurring over 10 years in Central Finland Ann Chir Gynaecol 1999 88 1 55 60 10230684

[B19] Sund R Utilisation of administrative registers using scientific knowledge discovery Intelligent Data Analysis 2003 7 6 501 519 http://iospress.metapress.com/openurl.asp?genre=article&issn=1088-467X&volume=7&issue=6&spage=501

[B20] Powell AE Davies HT Thomson RG Using routine comparative data to assess the quality of health care: understanding and avoiding common pitfalls Qual Saf Health Care 2003 12 2 122 128 10.1136/qhc.12.2.122 12679509 PMC1743685

[B21] Gissler M Haukka J Finnish health and social welfare registers in epidemiological research Norsk Epidemiologi 2004 14 1 113 120 http://www.ntnu.no/ojs/index.php/norepid/article/viewFile/284/262

[B22] Sund R Nurmi-Lüthje I Lüthje P Tanninen S Narinen A Keskimäki I Comparing properties of audit data and routinely collected register data in case of performance assessment of hip fracture treatment in Finland Methods of Information in Medicine 2007 46 5 558 566 17938779 10.1160/me0382

[B23] Sund R Methodological perspectives for register-based health system performance assessment. Developing a hip fracture monitoring system in Finland. STAKES research report 174 2008 Helsinki: National Research and Development Centre for Welfare and Health http://urn.fi/URN:ISBN:978-951-33-2132-1

[B24] Jiang HX Majumdar SR Dick DA Moreau M Raso J Otto DD Johnston DW Development and initial validation of a risk score for predicting in-hospital and 1-year mortality in patients with hip fractures J Bone Miner Res 2005 20 3 494 500 10.1359/JBMR.041133 15746995

[B25] Roos LL Walld RK Romano PS Roberecki S Short-term mortality after repair of hip fracture. Do Manitoba elderly do worse? Med Care 1996 34 4 310 326 10.1097/00005650-199604000-00003 8606556

[B26] Sund R Liski A Quality effects of operative delay on mortality in hip fracture treatment Qual Saf Health Care 2005 14 5 371 377 10.1136/qshc.2004.012831 16195573 PMC1744067

[B27] Foss NB Kehlet H Mortality analysis in hip fracture patients: implications for design of future outcome trials Br J Anaesth 2005 94 1 24 29 10.1093/bja/aei010 15516350

[B28] Heikkinen T Jalovaara P Four or twelve months' follow-up in the evaluation of functional outcome after hip fracture surgery? Scand J Surg 2005 94 1 59 66 15865120 10.1177/145749690509400115

[B29] Laukkanen P Karppi P Heikkinen E Kauppinen M Coping with activities of daily living in different care settings Age Ageing 2001 30 6 489 494 10.1093/ageing/30.6.489 11742778

[B30] Normand S-LT Glickman ME Gatsonis CA Statistical methods for profiling providers of medical care: issues and applications J Am Stat Assoc 1997 92 439 803 814 10.2307/2965545

[B31] DeLong ER Peterson ED DeLong DM Muhlbaier LH Hackett S Mark DB Comparing risk-adjustment methods for provider profiling Stat Med 1997 16 23 2645 2664 10.1002/(SICI)1097-0258(19971215)16:23<2645::AID-SIM696>3.0.CO;2-D 9421867

[B32] Goldstein H Spiegelhalter D League tables and their limitations: Statistical issues in comparisons of institutional performance (with discussion) J R Stat Soc Ser A Stat Soc 1996 159 3 385 443 10.2307/2983325

[B33] Marshall EC Spiegelhalter DJ Leyland AH, Goldstein H Institutional performance Multilevel modelling of health statistics 2001 Chichester: John Wiley & Sons 127 142

[B34] Burgess JF Jr Christiansen CL Michalak SE Morris CN Medical profiling: improving standards and risk adjustments using hierarchical models J Health Econ 2000 19 3 291 309 10.1016/S0167-6296(99)00034-X 10977193

[B35] Törnqvist L Vartia P Vartia YO How should relative changes be measured? Am Stat 1985 39 1 43 46 10.2307/2683905

[B36] Christian CK Gustafson ML Betensky RA Daley J Zinner MJ The Leapfrog volume criteria may fall short in identifying high-quality surgical centers Ann Surg 2003 238 4 447 455 Discussion 455-457 14530717 10.1097/01.sla.0000089850.27592.eb PMC1360105

[B37] Royston P Altman DG Regression using fractional polynomials of continuous covariates: Parsimonious parametric modelling Applied Statistics 1994 43 3 429 467 10.2307/2986270

[B38] Gurrin LC Scurrah KJ Hazelton ML Tutorial in biostatistics: spline smoothing with linear mixed models Stat Med 2005 24 21 3361 3381 10.1002/sim.2193 16206247

[B39] Crainiceanu CM Ruppert D Wand MP Bayesian analysis for penalized spline regression using WinBUGS Journal of Statistical Software 2005 14 14 http://www.jstatsoft.org/v14/i14

[B40] Brooks SP Gelman A Alternative methods for monitoring convergence of iterative simulations J Comput Graph Stat 1998 7 434 455 10.2307/1390675

[B41] Spiegelhalter D Best NG Carlin BP van der Linde A Bayesian measures of model complexity and fit (with discussion) J R Stat Soc Ser B 2002 64 583 639 10.1111/1467-9868.00353

[B42] Rissanen P Sund R Linna M Idänpään-Heikkilä U Rousi T Nordback I Does hospital volume influence the effectiveness of hip fracture treatments? [In Finnish] Suomen Lääkärilehti 2003 58 12 1419 1423 http://www.laakarilehti.fi/sisallys/index.html?nr=12,yr=2003

[B43] Flood AB Scott WR Ewy W Does practice make perfect? Part I: The relation between hospital volume and outcomes for selected diagnostic categories Med Care 1984 22 2 98 114 10.1097/00005650-198402000-00002 6700280

[B44] Riley G Lubitz J Outcomes of surgery among the Medicare aged: surgical volume and mortality Health Care Financ Rev 1985 7 1 37 47 10317676 PMC4191511

[B45] Maerki SC Luft HS Hunt SS Selecting categories of patients for regionalization. Implications of the relationship between volume and outcome Med Care 1986 24 2 148 158 10.1097/00005650-198602000-00006 3080647

[B46] Luft HS Hunt SS Maerki SC The volume-outcome relationship: practice-makes-perfect or selective-referral patterns? Health Serv Res 1987 22 2 157 182 3112042 PMC1065430

[B47] Hughes RG Garnick DW Luft HS McPhee SJ Hunt SS Hospital volume and patient outcomes. The case of hip fracture patients Med Care 1988 26 11 1057 1067 10.1097/00005650-198811000-00004 3185017

[B48] Burns LR Wholey DR The effects of patient, hospital, and physician characteristics on length of stay and mortality Med Care 1991 29 3 251 271 10.1097/00005650-199103000-00007 1997754

[B49] Hamilton BH Hamilton VH Estimating surgical volume-outcome relationships applying survival models: accounting for frailty and hospital fixed effects Health Econ 1997 6 4 383 395 10.1002/(SICI)1099-1050(199707)6:4<383::AID-HEC278>3.0.CO;2-L 9285231

[B50] Taylor HD Dennis DA Crane HS Relationship between mortality rates and hospital patient volume for Medicare patients undergoing major orthopaedic surgery of the hip, knee, spine, and femur J Arthroplasty 1997 12 3 235 242 10.1016/S0883-5403(97)90018-8 9113536

[B51] Hamilton BH Ho V Does practice make perfect? Examining the relationship between hospital surgical volume and outcomes for hip fracture patients in Quebec Med Care 1998 36 6 892 903 10.1097/00005650-199806000-00012 9630130

[B52] Lavernia CJ Hemiarthroplasty in hip fracture care: effects of surgical volume on short-term outcome J Arthroplasty 1998 13 7 774 778 10.1016/S0883-5403(98)90029-8 9802663

[B53] Wenning M Hupe K Scheuer I Senninger N Smektala R Windhorst T Does quantity mean quality? An analysis of 116,000 patients regarding the connection between the number of cases and the quality of results [in German] Chirurg 2000 71 6 717 722 10.1007/s001040051126 10948741

[B54] Smektala R Paech S Wenning M Hupe K Ekkernkamp A Does hospital structure influence the outcome of operative treatment of femoral neck fractures? [in German] Zentralbl Chir 2002 127 3 231 237 10.1055/s-2002-24247 11935489

[B55] Franzo A Francescutti C Simon G Risk factors correlated with post-operative mortality for hip fracture surgery in the elderly: a population-based approach Eur J Epidemiol 2005 20 12 985 991 10.1007/s10654-005-4280-9 16331429

[B56] Shah SN Wainess RM Karunakar MA Hemiarthroplasty for femoral neck fracture in the elderly surgeon and hospital volume-related outcomes J Arthroplasty 2005 20 4 503 508 10.1016/j.arth.2004.03.025 16124968

[B57] Gandjour A Weyler E-J Cost-effectiveness of referrals to high-volume hospitals: An analysis based on a probabilistic Markov model for hip fracture surgeries Health Care Manag Sci 2006 9 359 369 10.1007/s10729-006-0000-6 17186771

[B58] Liu JH Zingmond DS McGory ML SooHoo NF Ettner SL Brook RH Ko CY Disparities in the utilization of high-volume hospitals for complex surgery JAMA 2006 296 16 1973 1980 10.1001/jama.296.16.1973 17062860

[B59] Browne JA Pietrobon R Olson SA Hip fracture outcomes: Does surgeon or hospital volume really matter? J Trauma 2009 66 3 809 814 10.1097/TA.0b013e31816166bb 19276758

[B60] Forte ML Virnig BA Swiontkowski MF Bhandari M Feldman R Eberly LE Kane RL Ninety-day mortality after intertrochanteric hip fracture: Does provider volume matter? J Bone Joint Surg Am 2010 92 4 799 806 10.2106/JBJS.H.01204 20360501

[B61] Li Y Cai X Mukamel DB Glance LG The volume-outcome relationship in nursing home care. An examination of functional decline among long-term care residents Medical Care 2010 48 1 52 57 10.1097/MLR.0b013e3181bd4603 19890222 PMC9577586

[B62] Huusko TM Karppi P Avikainen V Kautiainen H Sulkava R Intensive geriatric rehabilitation of hip fracture patients: a randomized, controlled trial Acta Orthop Scand 2002 73 4 425 431 10.1080/00016470216324 12358116

[B63] Raivio M Korkala O Pitkala K Tilvis R Rehabilitation outcome in hip-fracture: Impact of weight-bearing restriction - A preliminary investigation Phys Occup Ther Geriatr 2005 22 4 1 9 10.1300/J148v22n04_01

[B64] Schneeweiss S Seeger JD Maclure M Wang PS Avorn J Glynn RJ Performance of comorbidity scores to control for confounding in epidemiologic studies using claims data Am J Epidemiol 2001 154 9 854 864 10.1093/aje/154.9.854 11682368

[B65] Sund R Riihimäki J Mäkelä M Vehtari A Lüthje P Huusko T Häkkinen U Modeling the length of the care episode after hip fracture: Does the type of fracture matter? Scand J Surg 2009 98 3 169 174 19919923 10.1177/145749690909800308

